# Evaluation and clinical significance of contrast-enhanced ultrasound on changes in liver blood flow perfusion after TIPS surgery

**DOI:** 10.1097/MD.0000000000037899

**Published:** 2024-04-26

**Authors:** Meirong Yang, Fei Qin, Yan Zhou, Yueping Yao, Zhonghua Lu, Wei Chen

**Affiliations:** a Department of Infectious Diseases, Wuxi Fifth People’s Hospital, Wuxi, China; b Department of Ultrasound, Wuxi Fifth People’s Hospital, Wuxi, China; c Department of Interventional, Wuxi Fifth People‘s Hospital, Wuxi, China.

**Keywords:** contrast-enhanced ultrasound, hepatic encephalopathy, liver blood flow perfusion, PSG, TIPS

## Abstract

To investigate the clinical value of contrast-enhanced ultrasound in the prediction of hepatic encephalopathy (HE) in patients with hepatitis B cirrhosis after intrahepatic portal-systemic shunt via jugular vein. In this retrospective study, we collected data from 75 patients with hepatitis B, cirrhosis, and portal hypertension who underwent jugular intrahepatic portosystemic shunt from February 2019 to February 2022. The diagnostic instrument used was the TOSHIBA Aplio500 color Doppler ultrasound with contrast-enhanced ultrasound capabilities. The trial group comprised 20 patients with HE within 3 months postsurgery, while the control group (CG) included 55 patients without HE within the same postoperative period. All patients underwent various examinations before and within 48 hours after surgery, including observation of liver and spleen size and stent position, as well as assessment of blood flow direction in portal and hepatic veins. Subsequently, contrast-enhanced ultrasound was employed to examine and observe perfusion changes of contrast agents in hepatic veins, hepatic arteries, and portal veins (PV). Changes in PV pressure gradient, intrahepatic, and stent blood flow perfusion (BFP) were explored in both postoperative trials and CGs. The trial group exhibited higher BFP volume, PV pressure gradient difference, and percentage decrease compared to the CG. A weak positive correlation was observed between blood flow within the liver stent and PV pressure gradient difference, as well as the percentage decrease in PV pressure gradient. The correlation coefficient between blood flowing perfusion volume within the stent and the difference in PV pressure gradient was *R* = 0.415 (*P* = .000). The correlating coefficient between BFP amount within the stent and the percentage decrease in PV pressure gradient was *R* = 0.261 (*P* = .027). The area under the receiver operating characteristic curve for stent perfusion volume, difference in PV pressure gradient, and percentage decrease in PV pressure gradient was 0.691, 0.759, and 0.742, respectively. An increase in PV pressure gradient accelerates blood flow within the stent, predisposing to HE. Changes in hepatic BFP following transjugular intrahepatic portosystemic shunt can effectively predict the occurrence of HE, demonstrating significant clinical relevance.

## 1. Introduction

Transjugular intrahepatic portosystemic shunt (TIPS) is currently a widely employed method for treating portal hypertension (PH) and its complications in liver cirrhosis (LC).^[1,2]^ Common complications following TIPS surgery include hepatic encephalopathy (HE) and stent dysfunction.

In patients with LC and PH, as the condition worsens, the perfusion volume of the portal vein (PV) significantly decreases, resulting in substantial changes in liver hemodynamics and leading to HE.^[3]^ Literature records indicate that approximately 20% to 31% of patients experience HE after TIPS, with the main inducement being the direct introduction of part of PV blood flow to the inferior vena cava without passing through the liver. This leads to an increase in blood ammonia, resulting in elevated blood ammonia in the brain, further inducing brain tissue edema and disorders, ultimately culminating in HE.^[4,5]^ Studies have demonstrated that when the portosystemic gradient (PSG) drops below 12 mm Hg, the occurrence of gastroesophageal vein rupture is nearly nonexistent. However, the Liver Disease Association has highlighted a positive correlation between PSG decrease and the risk of inducing HE. A greater decrease in PSG leads to increased blood flow during stent shunting, thereby elevating the risk of HE.^[6]^ Consequently, it is essential to analyze changes in liver blood flow perfusion (BFP) in patients post-TIPS.

Scholars have found that the combination of color Doppler flow imaging (CDFI) technology and traditional ultrasound technology can indirectly reflect stent patency and blood flow but is prone to false positive results.^[6]^ To address this, the study proposes the use of contrast-enhanced ultrasound (CEUS) technology, which enables the visual display of BFP in the liver and the direct observation of the complete process of contrast agent passage through different veins and the stent. It is anticipated that the relationship between TIPS and the incidence of HE can be effectively diagnosed using this approach.****

## 2. Materials and methods

### 2.1. General materials

**Figure 1.**
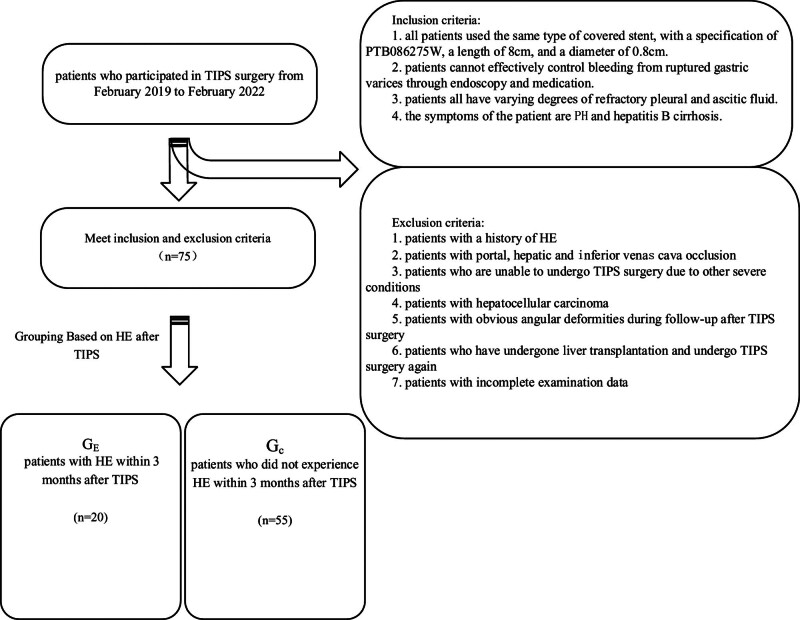
Flowchart for choosing the study population.

The study was approved by the Ethics committee of Wuxi Fifth People’s Hospital. The experiment collected hepatitis B cirrhosis patients who participated in TIPS surgery from February 2019 to February 2022. Seventy-five patients with LC and PH who met the criteria were selected, and 20 patients with HE within 3 months after TIPS surgery were assigned to the trial group (TG). Among them, 14 were male and 6 were female, aged between 40 and 75 years old, whose mean age is 60.14 ± 12.20 years old. Fifty-five patients who did not experience HE within 3 months after TIPS surgery were set as the control group (CG), with 42 males and 13 females, aged between 28 and 72 years old, whose mean age is 55.06 ± 12.49 years old. All patients undergo a series of routine examinations before and within 48 hours after TIPS surgery, including blood ammonia, liver and kidney examination, color Doppler ultrasound examination, and blood routine. Patient follow-up was conducted within 3 months after TIPS surgery, and if the patient developed HE, the experiment was discontinued. The experiment used the Delirium Scale to score patients on HE, based on the staging of HE, degree of ascites, time of prothrombin, albumin, and serum bilirubin. Among them, the score for Levels A, B, and C is 5–6, 7–9, and 10–15 points, respectively^[7]^ (Fig. 1).

All patients underwent a series of routine examinations before and within 48 hours after TIPS surgery. These examinations included blood ammonia, liver and kidney assessments, color Doppler ultrasound examinations, and blood routine tests. Patient follow-up occurred within 3 months post-TIPS surgery, and if a patient developed HE, the experiment was discontinued. The Delirium Scale was used to score patients on HE based on the staging of HE, degree of ascites, time of prothrombin, albumin, and serum bilirubin. The scores for Levels A, B, and C were 5–6, 7–9, and 10–15 points, respectively.^[7]^

The inclusion criteria for participants in this experiment comprised 4 points: first, all patients used the same type of covered stent (PTB086275W) with a length of 8 cm and a diameter of 0.8 cm. Second, patients were unable to effectively control bleeding from ruptured gastric varices through endoscopy and medication. Third, patients exhibited varying degrees of refractory pleural and ascitic fluid. Fourth, patients presented symptoms of PH and hepatitis B cirrhosis. Exclusion criteria included 6 main points: first, patients with a history of HE. Second, patients with portal, hepatic, and inferior vena cava occlusion. Third, patients are unable to undergo TIPS surgery due to other severe conditions. Fourth, patients with hepatocellular carcinoma. Fifth, patients with obvious angular deformities during follow-up after TIPS surgery. Sixth, patients who underwent liver transplantation and subsequently underwent TIPS surgery again. Seventh, patients with incomplete examination data.^[8,9]^

### 2.2. Instruments and methods

The diagnostic instrument used in this experiment is TOSHIBA Aplio500 color Doppler ultrasonic testing with CEUS function, its center frequency is 3.5 MHz, and the broadband convex array probe is used. The contrast agent used in the experiment is Sonovue (Brocca Milan Italy), which is packaged as a freeze-dried powder. During use, 5 mL of physiological saline is injected to dissolve it and then shaken vigorously to form a suspension for later use.^[10,11]^

**Figure 2.**
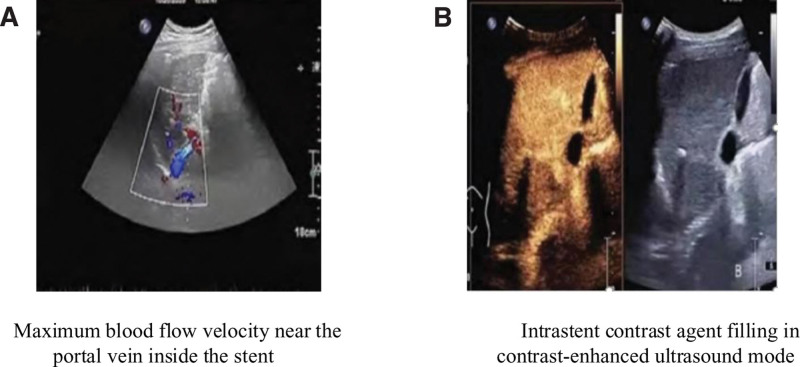
Color Doppler ultrasound and contrast-enhanced ultrasound examination.

Before TIPS surgery, routine CEUS examination is performed. In CEUS imaging, it is necessary to train patients to hold their breath and set the imaging mode to contrast pulse sequence mode. Contrast (1.2 mL) agent was injected through superficial vein of elbow, and then 5 mL of normal saline was rapidly injected. After 5 seconds, the patient was informed to hold his breath and reduce the respiratory rate. If these imaging results do not meet the requirements, it is necessary to wait until the previous imaging agent is completely metabolized before injection. The next injection time is about 10 minutes later. Before performing CEUS, it is necessary to explain to the patient the contraindications of injecting contrast agents and the potential adverse reactions that may occur in the body after the contrast and to sign an informed consent form.^[12]^ Figure 2 shows color Doppler ultrasound and CEUS examination.

Next, TIPS surgery was performed according to the guidelines, and the entire process was completed by experienced senior doctors in the interventional department. Routine disinfection should be performed on the right internal jugular vein puncture field and Femoral artery of the patient before operation. After towel laying, patient’s right femoral artery was punctured under local anesthesia to determine the position of PV’s left and right branches, and then the right internal jugular vein was punctured under local anesthesia. Then, guided by a guide wire, a puncture needle was inserted into PV through the dilated duct and puncture pathway. Then, guided by an extended and hard guide wire, it enters a single bend through a sheath tube and then enters the splenic vein for PV angiography. The catheter was then retracted to the opening of inferior vena cava and the shunt was dilated with a balloon. After a long sheath was inserted into the main PV, the guide wire was removed. Then PV pressure was measured through the catheter. Subsequently, TIPS stent was inserted along the guide wire. After positioning, the stent was completely released. Throughout the entire process, a balloon was used to expand the shunt. PV angiography was performed again, and the shunt blood flow was unobstructed. The blood flow of TIPS shunt was flowing to inferior vena cava. The stent position was reasonable, and no contrast agent overflowed. Finally, the pressure in PV was measured again. After the operation, the right femoral artery and internal jugular venipuncture puncture points were pressurized and banded.^[13,14]^

During TIPS, CEUS was performed on PV before stent release, and then the pressure of PV and inferior vena cava was measured and recorded. When stent is released, CEUS should be performed on PV again to observe stent and PV’s situation. And PV and inferior vena cava pressure should be measured and recorded. All patients’ surgeries showed that stent had been successfully implanted, and ultrasound imaging showed that stent was in an unobstructed state, without complications such as stent heterotopic, twisting, or “cap” stenosis. The success of TIPS surgery is marked by the successful establishment of a shunt and a decrease in the pressure gradient of PV below 12 mm Hg. After TIPS, liver protection or drug treatment shall be carried out according to the postoperative reaction of each patient. If the patient has cognitive and behavioral abnormalities and other HE symptoms, it is necessary to limit the protein intake of the patient and supplement branched-chain amino acids. In serious cases, enema can be carried out.^[15]^

### 2.3. Statistical methods

The experiment was organized and statistically analyzed by SPSS 22.0, using mean ± standard deviation to represent the measurement data. Paired data were analyzed using paired *t* tests, and counting data were represented by case number or percentage. Two groups’ comparison was conducted using *χ*^2^ test and independent-sample *t* test. When *P* < .05, the data comparison representing inter-group comparison has statistical significance.

## 3. Research results

### 3.1. Compilation and analysis of patient clinical data

The study initially conducted a statistical analysis of the basic clinical information of the 2 patient groups, as presented in Table 1. The *P* values for age, gender, and HE score in both the trial group (TG) and the CG were all >.05. Specifically, the *P* value for gender was 0.742, for age was 0.314, and for HE score, the *P* values were .859, .743, and .215 in the order of A to C, respectively. These results indicate that there were no statistically significant differences in age, gender, or HE score between the 2 groups. Meanwhile, the blood ammonia value of TG was significantly higher than CG with a *P* value of .000.

**Table 1:** Patient clinical data analysis.

Information	TG	CG	*P*	Statistic
Gender: male/female	14 (70%)/6 (30%)	42 (76%)/13 (24%)	.742	*χ* = 0.115
Age	60.14 ± 12.20	55.06 ± 12.49	.314	*t* = 1.011
Liver function				
A	3 (15%)	7 (13%)	.859	*χ* = 0.276
B	8 (40%)	20 (36%)	.743	*χ* = 0.125
C	9 (45%)	28 (51%)	.215	*χ* = 0.186
Blood ammonia	98 ± 33.83	49 ± 12.78	.000	*t* = 5.547

CG = control group, TG = trial group.

### 3.2. Analyzing hemodynamic detection results

This experiment conducted statistical analysis on 2 groups’ hepatic artery, splenic vein, and PV after TIPS surgery. The evaluation and analysis indicators include hepatic artery velocity (HAV), inner diameter of hepatic artery (HAD), splenic vein velocity (SVV), internal diameter of the splenic vein (SVD), PV flow rate (PVV), and PV′ internal diameter (PVD). Table 2 shows the analysis results of various indicators before and after TIPS surgery for 2 groups’ patients. *P* values of HAV, HAD, SVV, PVV, and PVD indicators before and after TIPS surgery in both groups of patients were >.05. *P* values of HAV, HAD, SVV, PVV, and PVD indicators before TIPS surgery in 2 groups’ patients were .149, .688, .439, .247, and .537, respectively. *P* values of HAV, HAD, SVV, PVV, and PVD indicators after TIPS surgery in both groups’ patients were .914, .264, .316, .472, and .177, respectively. This indicates that HAV, HAD, SVV, PVV, and PVD indicators before and after surgery are the same. Meanwhile, after TIPS surgery, SVD of TG was lower than CG, and its *P* was .04, <.05 (Table 2).

**Table 2:** Analysis of hepatic artery, splenic vein, and PV after TIPS.

Index	TG	CG	Statistic	*P*
HAV (cm/s)				
Preoperative	90.68 ± 30.29	80.71 ± 25.18	*t* = 1.460	.149
Postoperative	102.04 ± 36.89	103.16 ± 41.73	*t* = −1.108	.914
HAD (mm)				
Preoperative	3.53 ± 1.02	3.62 ± 0.89	*t* = −0.403	.688
Postoperative	3.76 ± 0.99	3.50 ± 0.87	*t* = −1.126	.264
SVV (cm/s)				
Preoperative	18.50 ± 4.97	19.47 ± 4.84	*t* = −0.779	.439
Postoperative	25.91 ± 7.39	28.12 ± 9.03	*t* = −1.010	.316
SVD (mm)				
Preoperative	10.95 ± 3.63	12.09 ± 3.97	*t* = −1.158	.251
Postoperative	8.84 ± 2.04	10.49 ± 2.45	*t* = −2.762	.004
PVV (cm/s)				
Preoperative	13.27 ± 3.73	14.33 ± 3.49	*t* = −1.167	.247
Postoperative	29.32 ± 9.88	27.78 ± 7.66	*t* = −0.724	.472
PVD (mm)				
Preoperative	15.14 ± 2.32	15.15 ± 3.45	*t* = −0.620	.537
Postoperative	11.25 ± 1.89	12.02 ± 2.30	*t* = −1.363	.177

HAD = inner diameter of hepatic artery, HAV = hepatic artery velocity, SVD = diameter of the splenic vein, SVV = splenic vein velocity, PV = portal vein, PVV = PV flow rate, PVD = PV′ internal diameter, TIPS = transjugular intrahepatic portosystemic shunt.

Table 3 displays the analysis results of BFP in the hepatic artery, splenic vein, PV, and stent following TIPS surgery. The stent perfusion volume in the TG was notably higher than that in the CG, with a *P* value for stent perfusion flow in both groups of patients being 0.04, which indicates statistical significance. On the other hand, the *P* values for splenic vein, hepatic artery, and PV blood flow before and after surgery were all >.05. Specifically, the preoperative *P* were .612, .547, and .216, respectively. The postoperative *P* were .922, .257, and .216, respectively. These results suggest that there were no significant differences in blood flow in the splenic vein, hepatic artery, and PV before and after surgery, these indicate consistency in blood flow differences in these vessels.

**Table 3:** Analysis of hepatic artery, splenic vein, and PV after TIPS.

Index	TG	CG	Statistic	*P*
Intrastent BFP volume (mL/min)	2098.56 ± 652.90	674.61 ± 507.11	*t* = 2.999	.004
Splenic venous flow (mL/min)				
Preoperative	650.53 ± 447.71	857.51 ± 619.01	*t* = 0.515	.612
Postoperative	570.90 ± 311.37	855.56 ± 434.37	*t* = 0.098	.922
Hepatic artery flow (mL/min)				
Preoperative	336.73 ± 272.31	303.72 ± 183.88	*t* = 0.605	.547
Postoperative	411.40 ± 239.81	350.41 ± 195.31	*t* = 1.142	.257
Portal venous flow (mL/min)				
Preoperative	837.28 ± 321.15	971.88 ± 345.62	*t* = −1.247	.216
Postoperative	81027.34 ± 480.03	1115.62 ± 505.93	*t* = −0.694	.490

BFP = blood flow perfusion, PV = portal vein, TIPS = transjugular intrahepatic portosystemic shunt.

Table 4 shows the comparison of the diameter and maximum blood flow velocity of splenic and PVs before and after TIPS surgery in all patients. At each postoperative time point, compared to before surgery, the inner diameters of splenic vein and PV decreased (*P > *.05). And the maximum blood flow velocity of splenic vein and PV increased after surgery (*P > .*05).

**Table 4:** Comparison of the diameter and maximum blood flow velocity of splenic and PVs before and after surgery.

	Preoperative	Postoperative	*P*
Splenic vein inner diameter (mm)	12.15 ± 1.24	10.84 ± 2.11	.264
Splenic vein flow rate (cm/s)	16.20 ± 4.31	24.06 ± 2.17	.548
PV diameter (mm)	14.79 ± 1.28	13.85 ± 1.06	.357
PV flow rate (cm/s)	20.18 ± 0.99	27.37 ± 8.01	.639

PV = portal vein.

### 3.3. Analysis of preoperative and postoperative PSG values, PSG differences, and percentage decrease in PSG between TG and CG in TIPS

**Figure 3.**
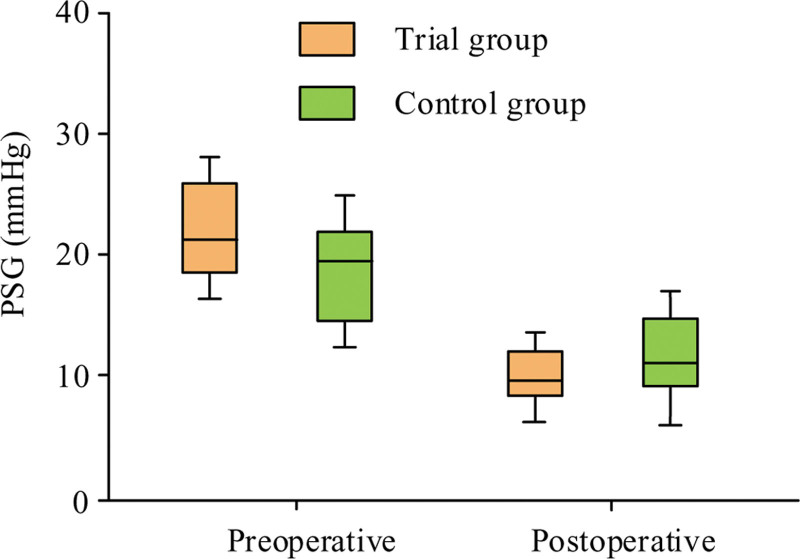
Box results of preoperative and postoperative PSG in the observation group and CG. CG = control group, PSG = portosystemic gradient.

The study further explored the preoperative and postoperative PSG values, PSG differences, and percentage decrease in PSG between TG and CG in TIPS. Figure 3 shows the box plots of PSG values before and after surgery for 2 groups. Before TIPS surgery, the PSG values of these 2 groups were 22.42 ± 4.36 and 20.62 ± 3.34, respectively. The postoperative PSG values were 9.69 ± 2.38 and 10.43 ± 3.64, respectively. After surgery, PSG values of both groups decreased.

**Figure 4.**
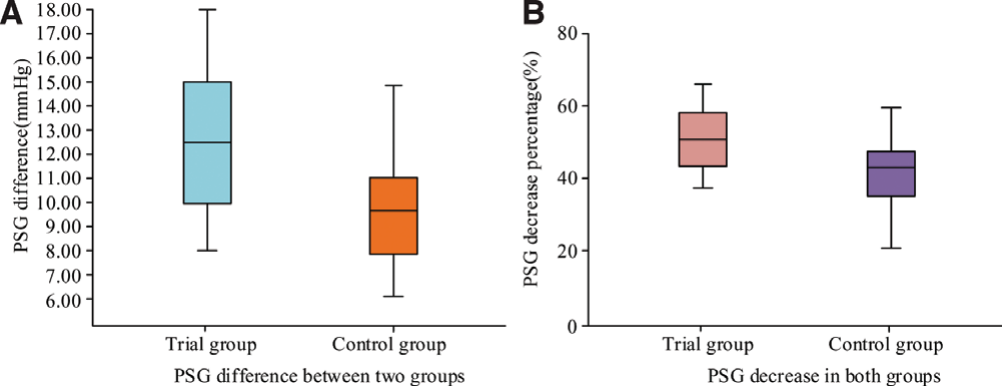
Box results of PSG difference and PSG reduction percentage. PSG = portosystemic gradient.

Figure 4 shows the box plot of the difference and decreasing percentage in PSG between these 2 groups after surgery. The PSG difference and PSG decreasing percentage of TG are greater than CG. Among them, the PSG difference of TG is 12.48 ± 3.15, and decreasing percentage in PSG is 51.57 ± 8.37. The PSG difference of CG is 9.57 ± 2.38, and decreasing percentage in PSG is 43.63 ± 8.46. The decrease in PSG of TG is greater.

Table 5 presents the statistical analysis of PSG values, PSG differences, and percentage decrease in PSG before and after TIPS surgery. *P* values of preoperative and postoperative PSG for TG and CG were .059 and .115, respectively, both >.05, indicating no statistical significance. Meanwhile, the difference and decreasing percentage in PSG between these 2 groups’ patients were *P* = .000, both <.05.

**Table 5:** Statistical analysis of preoperative and postoperative PSG values, PSG differences, and percentage decrease in PSG in TIPS.

Index (mm Hg)	TG	CG	Statistic	*P*
Preoperative PSG	22.42 ± 4.36	20.62 ± 3.34	*t* = 1.916	.059
Postoperative PSG	9.69 ± 2.38	10.43 ± 3.64	*t* = −1.587	.115
PSG difference	12.48 ± 3.15	9.57 ± 2.38	*t* = 4.120	.000
Percentage decrease (%)	51.57 ± 8.37	43.63 ± 8.46	*t* = 3.854	.000

CG = control group, PSG = portosystemic gradient, TG = trial group.

### 3.4. Relationship between Intrastent BFP, PSG, and HE

**Figure 5.**
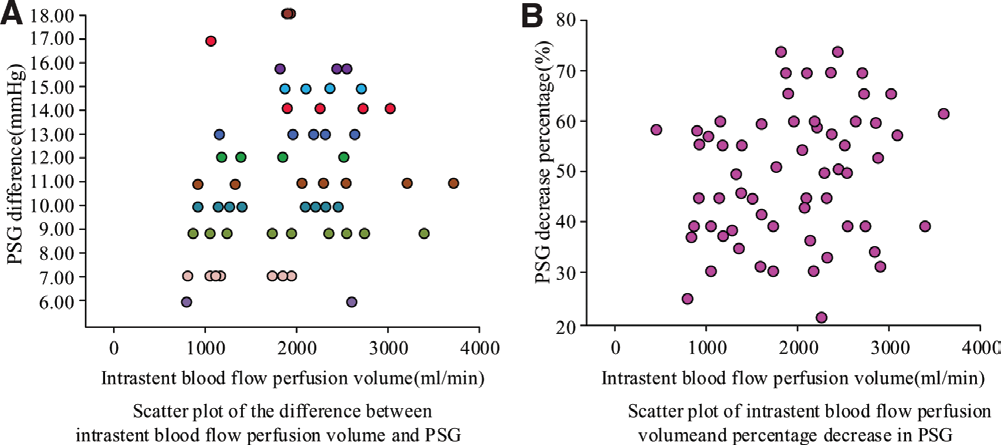
Scatter plot of the relationship between hepatic BFP volume, PSG difference, and PSG decreasing percentage. BFP = blood flow perfusion, PSG = portosystemic gradient.

Figure 5 shows the scatter plot of the relationship between BFP volume inside stent and PSG difference, as well as decreasing percentage in PSG. There is a weak positive correlation between the BFP volume inside stent and PSG difference, with a correlation coefficient of *R* = 0.415 and *P* = .000. There is a weak positive correlation between the amount of BFP inside stent and the percentage of PSG decreasing, with *R* = 0.261 and *P* = .027.

**Figure 6.**
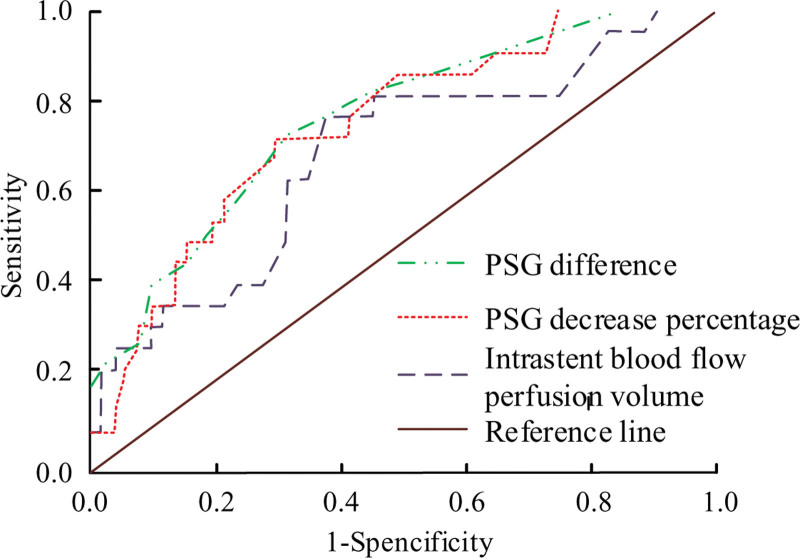
Intrastent BFP volume, PSG difference, PSG decreasing percentage and receiver operating characteristic of hepatic encephalopathy. BFP = blood flow perfusion, PSG = portosystemic gradient.

Figure 6 shows the receiver operating characteristics (ROC) curve of BFP volume, PSG difference, and HE within stent. The ROC area under intrastent BFP volume is 0.691, the area under PSG difference is 0.759, and the area under PSG decreasing percentage is 0.742.

Table 6 shows the ROC analysis of invasive blood flow fusion volume, PSG difference, PSG decreasing percentage, and HE. The sensitivity and specificity of detecting HE in TG by ROC are both higher than 60%. It indicates that measuring hepatic BFP can effectively predict the risk of HE occurrence.****

**Table 6:** Receiver operating characteristic analysis.

	AUC	Sensitivity	Specificity
PSG difference	0.759	0.72	0.68
Percentage decrease	0.742	0.67	0.71
Intrastent BFP volume	0.691	0.78	0.62

BFP = blood flow perfusion, PSG = portosystemic gradient.

## 4. Discussion

TIPS surgery mainly constructs an artificial channel by planting a stent between PV and Inferior vena cava, which can effectively treat patients with cirrhosis and PH.^[16]^ Research has shown that TIPS surgery can make PV pressure reduced, but postoperative HE occurring is significantly increased. Meanwhile, although TIPS surgery has significant short-term efficacy, in the later stages of recovery, patients are highly likely to experience complications such as stent stenosis, occlusion, and HE. Therefore, it is necessary to monitor the patency of the stent and changes in intrahepatic blood flow in a timely manner in the early stage after TIPS surgery.^[17]^ Hussain et al^[18]^ found that CDFI technology can indicate the position of splenic vein, PV, hepatic artery, and stent, and CDFI can detect the direction and velocity of blood flow inside the stent. Researchers combine this technology with traditional ultrasound technology to indirectly reflect the patency of the stent, but this has significant limitations and low detection accuracy. And due to the difficulty of CDFI in detecting blood flow with low flow rates, it is likely that the normally unobstructed stent will be detected as occluded, resulting in false positive results. This study proposes a CEUS technology, which can visually display BFP in liver and directly observe the complete process of contrast agent passing through the hepatic vein, PV, hepatic artery, and stent. CEUS can intuitively reflect changes in blood flow within liver, and has high sensitivity and specificity for stent stenosis or occlusion, thus accurately diagnosing the patency of the stent. It can reflect the correlation between BFP volume in intrahepatic stent and PSG, PSG difference, as well as PSG decreasing percentage, thereby diagnosing HE occurring probability.^[19]^ However, opinions on PSG are not consistent, and studies have confirmed that The PSG reduction by one-third may reduce the risk of HE. In this study, it was found that the stent blood flow was weakly positively correlated with the percentage of PSG after TIPS and the decrease of PSG after TIPS. The more obvious the decrease of PSG, the faster the instantaneous blood flow rate, and the greater the possibility of HE occurrence. Other existing studies have also confirmed that decreased PSG can increase the risk of HE and liver function damage, and have achieved good clinical results.^[20,21]^ The conclusion in this article is still trustworthy, the changes of hepatic BFP after TIPS can effectively predict HE occurrence, and it has certain clinical significance. There is still no suitable criterion for in PSG. This is also the direction of our future research.

The study concluded its experimental investigation 3 months after TIPS surgery, as the period of HE usually occurs within 3 months after TIPS surgery.^[22]^ At the same time, due to the rapid increase in brain blood flow of human organs in the short term after surgery, it is easy to cause HE. Research has found that the pathogenesis of HE has not yet been fully determined. The current theories include pseudo-neurotransmitters theory, γ-aminobutyric acid theory, the theory of ammonia poisoning, manganese poisoning hypothesis, and glial disease hypothesis. Among them, the ammonia poisoning theory suggests that approximately 80% of ammonia in the body is converted into glutamine and urea through the circulation of ornithine in liver. However, when liver function is severely damaged, the ability of liver to metabolize ammonia will be significantly reduced, and the probability of ammonia entering the brain tissue will be greater, thus blocking citric acid cycle, resulting in neurological symptoms such as consciousness disorders. This study’s results are similar to these findings. That is, TG’s blood ammonia levels were higher than CG’s obviously, possibly due to the severe impact of high blood ammonia on the central nervous system, thereby inducing HE.

The study concludes at 3 months post-TIPS surgery, aligning with the typical timeframe for HE occurrence. Due to the rapid increase in brain blood flow shortly after surgery, the risk of HE is elevated. The pathogenesis of HE remains incompletely determined, with theories including pseudo-neurotransmitters, γ-aminobutyric acid, ammonia poisoning, manganese poisoning, and glial disease. The ammonia poisoning theory suggests that liver damage reduces ammonia metabolism, allowing its entry into the brain, disrupting the citric acid cycle, and causing neurological symptoms. This study’s results, indicating higher blood ammonia levels in the TG, align with these findings, potentially inducing HE due to the severe impact of elevated blood ammonia on the central nervous system. A weak positive correlation between intrastent BFP and PSG parameters is identified. Faster PSG decrease post-TIPS surgery results in a faster stent flow, increasing HE risk. Routine follow-up examinations should prompt intervention if PSG significantly decreases and stent flow is rapid.^[23]^ Research by Shoreibah et al^[24]^ supports increased PV perfusion after TIPS surgery, potentially alleviating liver function damage. This study corroborates increased PV blood flow post-TIPS surgery in both TG and CG. Higher pre-TIPS surgery PSG indicates a greater likelihood of bleeding symptoms. While efforts to minimize PSG during surgery are essential, a larger PSG decrease increases stent flow, elevating HE risk. Patients with a 10 mm stent diameter are more prone to HE than those with an 8 mm diameter. Since TIPS stent diameter is unchangeable, predicting HE occurrence is reliant on monitoring stent blood flow.^[25]^ Related studies have found that the occurrence of HE after TIPS surgery is closely related to PV shunting, but effective technical measures to prevent HE have not yet emerged. It has important medical value for early prevention, diagnosis, treatment, and evaluation of HE. This study indicates that age, shunt flow, and liver function are important factors for the occurrence of HE after TIPS surgery. Elderly patients over 65 years old are more prone to HE, possibly due to the higher sensitivity of the central nervous system to ammonia and aminobutyric acid neurotransmitters in the elderly population. Meanwhile, the liver function is poorer, its ability to metabolize ammonia is weaker, and patients are more prone to HE.^[26]^

In this study, there was a weak positive correlation between the changes in BFP volume inside stent after TIPS surgery and the difference and decreasing percentage in PSG. The correlation coefficient between invasive blood flow fusion volume and PSG difference is *R* = 0.415, *P* = .000. The correlation coefficient between invasive blood flow fusion volume and the percentage of PSG decrease is *R* = 0.261, *P* = .027. This indicates that the BFP of intrahepatic stent corresponds to a decrease in PSG in PV system. The decrease is greater in PSG, the blood flow diverted into systemic veins is greater. Changes detecting in PSG and hepatic BFP within stent are of great significance for prevention and treatment of HE.

The disadvantage of this study is that the stent used in this experiment is the same ideal TIPS stent, which has no obvious angular deformity or “cap” stenosis. In fact, this stent is not completely unobstructed, and the diameter of blood flow inside stent is uncertain, making it unsuitable for patients who do not meet this requirement to apply the results obtained in this study. And following-up rate’s loss in this experiment showed high level, and no hidden HE patients were detected. In addition, this study has small studying cases. In the future, large-sample multicenter experiments are needed.

In summary, TIPS surgery can make patients with cirrhosis and PH treated effectively, but the incidence of postoperative HE is relatively high. The postoperative invasive blood flow fusion volume detected by CEUS shows a weak positive correlation with PSG difference and percentage decreasing. Meanwhile, when PSG decreasing greater, blood flowing velocity is faster inside stent, and HE occurring risk is higher. Therefore, using CEUS technology to explore the impact of changes in liver BFP after TIPS on HE has certain clinical significance.

## Author contributions

**Conceptualization:** Meirong Yang, Fei Qin, Zhonghua Lu, Wei Chen.

**Data curation:** Fei Qin, Yan Zhou.

**Formal analysis:** Meirong Yang, Fei Qin, Yueping Yao.

**Investigation:** Fei Qin, Yueping Yao, Zhonghua Lu.

**Methodology:** Meirong Yang, Yan Zhou.

**Supervision:** Wei Chen.

**Validation:** Yueping Yao.

**Writing—original draft:** Meirong Yang, Fei Qin.

**Writing—review & editing:** Meirong Yang, Fei Qin, Zhonghua Lu, Wei Chen.
